# Cognitive flexibility as a protective factor for empathy

**DOI:** 10.3389/fpsyg.2022.1064494

**Published:** 2023-01-17

**Authors:** Zhiwei Cai, Bing Qi

**Affiliations:** College of Education, Hebei University, Baoding, China

**Keywords:** cognitive flexibility, cognitive flexibility trait, flexibility in perspective-switching, trait empathy, state empathy

## Abstract

Although cognitive flexibility has always been considered essential to empathy, the relevant findings have been inconsistent. Inconsistent results may be because cognitive flexibility is a multi-level structure, while empathy is also a multilayer structure, and there are differences in how researchers define and measure cognitive flexibility. Therefore, the study explores the relationship between cognitive flexibility and empathy from a multi-dimensional perspective. This study involved 105 China students aged between 18 and 22 (*M* age = 20.26, SD = 2.00) who completed the Cognitive Flexibility Scale (cognitive flexibility trait, cognitive flexibility at the individual level), perspective-switching flexibility task (perspective-switching flexibility, cognitive flexibility at the cognitive level), the Interpersonal Reactivity Index scale (IRI, traits empathy), Multi-dimensional Empathy Test (state empathy), 2-back task (inhibitory control), and Stroop task (working memory). After controlling for additional variables, the results showed that: (1) Cognitive flexibility traits negatively predicted trait cognitive (IRI-PT) and affective empathy (IRI-EC). (2) The Other/Self perspective-switching flexibility negatively predicted the affective component of state empathy. (3) Cognitive flexibility traits and Other/Self perspective-switching flexibility negatively predicted empathy even after controlling for one of these. The study’s results suggested that cognitive flexibility negatively predicts empathy and is a protective factor for reducing the cost of empathy and promoting emotion regulation.

## 1. Introduction

Empathy refers to understanding and/or sharing the emotions of others (Preston and De Waal, 2002; Decety and Jackson, 2004), including both trait and state empathy (Song et al., 2019; Zhao et al., 2021). Trait Empathy is a relatively stable personality trait measured by offline self-report scales, such as the Basic Empathy Scale and Interpersonal Reactivity Index. State Empathy is a mental state or process that is relatively unstable, evaluated by participants in response to a specific stimulus, based on the situation, and measured through specific emotional situations, such as the Multi-dimensional Empathy Test. Empathy plays a fundamental role in social functioning, and it helps people to understand feelings and connect with others. People who experience other-oriented compassion likely engage in more prosocial behaviors that help reduce interpersonal conflict and promote peer relationships (Eisenberg et al., 2010; Boele et al., 2019). However, the inherent cognitive costs of empathy might motivate people to avoid empathy. The cognitive costs refer to the subjective effort involved in experience sharing (Cameron et al., 2019) and feeling compassion (Scheffer et al., 2022). The effort has been defined as “subjective intensification of mental and/or physical activity in the service of meeting some goal” (Inzlicht et al., 2018; Cameron et al., 2019). Furthermore, experiencing self-oriented personal distress (Davis, 1983; Batson, 2009; Tone and Tully, 2014) likely causes adverse reactions such as depression (Tone and Tully, 2014) or anxiety (Gambin and Sharp, 2018) to the negative emotions of others. Thus, it is of great practical significance to investigate the protective factors for reducing the cost of empathy and promoting emotion regulation.

In this respect, the cognitive system effectively processing information is particularly important. Because empathy not only requires interaction between cognitive systems (Decety, 2011; Bird and Viding, 2014; Heyes, 2018) and interaction with the external environment (Tone and Tully, 2014), these challenge the cognitive system. Extant research reveals that empathy involves at least two main components, is a bottom-up and top-down process (Heyes, 2018). The two components of empathy are ‘top-down’ cognitive and ‘bottom-up’ emotional empathy. The cognitive component of empathy involves recognizing others’ emotions and understanding others’ perspectives. The affective component of empathy refers to automatic and alternative responses to others’ emotions (Decety and Jackson, 2004), which might result in vicarious distress or compassion. Although the specific brain regions for cognitive and emotional empathy are still under discussion, more researchers support the view that cognitive empathy and emotional empathy differ on a neural level. For example, one review found that affective empathy engages the limbic system and basal ganglia, and cognitive empathy engages the dorsomedial prefrontal cortex and dorsolateral prefrontal cortex (Healey and Grossman, 2018). However, some researchers suggest that (1) affective empathy for vicarious pain (seeing someone else in pain), which involves the anterior/mid cingulate cortex and anterior insula; (2) cognitive empathy (mentalizing) involving the dorsomedial prefrontal cortex, ventromedial prefrontal cortex, temporoparietal junction, superior temporal sulcus, and temporal pole; (3) compassion involving the ventral striatum/nucleus accumbens, subgenual anterior cingulate cortex, and medial orbitofrontal cortex (for reviews, see Weisz and Zaki, 2018; Stevens and Taber, 2021). Recent magnetic resonance images studies have also reported greater thickness in the left orbitofrontal and ventrolateral frontal cortices, bilateral anterior cingulate, superior frontal and occipital areas in the high-cognitive empathic group, greater thalamic volume in the high-affective empathic group (Uribe et al., 2019). These findings explain why some people have impaired cognitive empathy and unimpaired emotional empathy (Lehmann et al., 2014) and vice versa. According to the integrated model of empathy and emotion regulation, the generation of empathy includes three sub-processes: the perceptual process that promotes the detection of emotional cues, the mimicry/embodiment process that triggers emotional resonance, and the cognitive process that inferences the experience of others and manages the interactive representations related to self/others. Empathy’s mimicry/embodiment process is mainly automatic and does not require complex cognitive involvement, but both perception and cognitive processes do (Thompson et al., 2019).

Cognitive flexibility is a property of the cognitive system: at the cognitive level is the interaction of different cognitive components (e.g., executive functions, attention, representations, perception, coordination of task parameters with goals); at the individual’s level is the interaction of cognition with and context, task demands and so on (Ionescu, 2012). People with higher cognitive flexibility have a better cognitive system. Previous work has shown that executive function as a component of cognitive flexibility contributes to empathy (Heyes, 2018). Executive function, a series of top-down processing processes (Diamond, 2013), is associated with a wide range of higher cognitive abilities. Inhibitory control of its sub-components helps to suppress stimuli unrelated to goals cues, and working memory helps to maintain information and goals. For example, the dual processing model assumes that empathy consists of two processing paths: bottom-up and top-down. Bottom-up processing systems automatically respond to stimuli in the context (e.g., emotional faces or body postures). While top-down processing systems rely on executive functions (Singer and Lamm, 2009) to receive the output of automated processing systems and evaluate automated responses (e.g., the priority of current events), and modulate according to motivation (e.g., behavioral response; Zaki, 2014; Wondra and Ellsworth, 2015), affected by executive function (Heyes, 2018). Yet research on the relationship between two different levels of cognitive flexibility and empathy is still lacking.

At the cognitive level, cognitive flexibility means faster information processing and better attention switching (Monsell et al., 2003). At the individual level, cognitive flexibility is a general cognitive tendency, which means people have the consciousness, willingness, and efficacy to make flexible choices in any situation (Martin and Rubin, 1995). Studies have found that people with high cognitive flexibility are more able to effectively use multiple emotional regulation strategies (Gao et al., 2021), while people with better emotional regulation ability are more able to reduce the impact of other’s emotions on themselves (Eisenberg and Fabes, 1992; Lockwood et al., 2013). At the same time, cognitively flexible people experienced lower levels of depression after adverse events (Wang and Liu, 2017). Thus, these results indicate that cognitive flexibility may reduce the cost of empathy and promote emotional regulation, playing a protective role for empathy.

However, previous research on the relationship between cognitive flexibility and empathy has the following shortcomings. First, debates about the relationship between cognitive flexibility and empathy still exist. A preschooler’s one-year longitudinal follow-up experiment found that cognitive flexibility did not predict state empathy (Zeng et al., 2021), and studies using eye-tracking techniques also found an insignificant relationship between cognitive flexibility and state empathy (Yan et al., 2021). However, a meta-analysis that included a broader age range of subjects, without distinguishing between trait and state empathy, found that cognitive flexibility positively predicted the cognitive component of empathy but not the affective component (Yan et al., 2020). The researchers did not find a predictive effect of cognitive flexibility on empathy may because most of the existing studies selected children with a low level of cognitive flexibility, whose cognitive flexibility is still in the development stage and at a low level (Buttelmann and Karbach, 2017). Second, most previous studies have defined cognitive flexibility and empathy from a single perspective. For example, studies define cognitive flexibility only at the cognitive level and empathy as a stable personality trait (Aliakbari et al., 2013; Godfrey et al., 2020), which ignores cognitive flexibility at the individual level and state empathy influenced by specific situations. In addition, cognitive flexibility tasks used by previous research rarely involve specific mental processes that produce empathy. For example, in a classic test of cognitive flexibility called the Trail Making Test (Reitan, 1992), in a paper-and-pencil version, the participants are required to connect numbers and letters randomly distributed on a piece of paper in order (Thoma et al., 2011). This task mainly examined the ability of the subjects to switch between numbers and letters sequentially and did not involve the ability to represent and infer the views of others and to switch between self and others (Bird and Viding, 2014), which contributes to empathy.

Bird and Viding (2014) suggest that people generate empathy through self-other switching. The mutual collaboration of situation understanding system, theory of mind system, mirror neuron system, affective cue classification system, emotion representation system, and self-other switch (Bird and Viding, 2014). Problems in self-other switch will affect the generation and representation of empathy (Baron-Cohen and Wheelwright, 2004). Similar to this process, perspective-switching flexibility is the flexibility shown in the process of the perspective switch when people complete the spatial perspective taking of themselves or others, including the self-others switching. Furthermore, during spatial perspective taking, people must represent and infer perspectives different from their own. Studies have found that taking others’ spatial perspectives is related to taking others’ psychological perspectives, initiating prosocial thinking, and other social cognition and decision-making processes, and it is the basis of cognitive and emotional perspective-taking (Erle and Topolinski, 2017). Meanwhile, perspective-switching flexibility is also vital for the affective component of empathy (Chiu and Yeh, 2018).

The current study focuses on two questions: First, is there a significant relationship between two kinds of levels of cognitive flexibility and empathy? Second, whether different levels of cognitive flexibility independently predict empathy? We selected college students as participants. At the individual level, cognitive flexibility was evaluated by the cognitive Flexibility scale (CFS). The adapted spatial perspective-taking task assessed cognitive flexibility at the cognitive level. The 2-back and Stroop tasks have been used successfully to assess inhibitory control (Miyake et al., 2000) and working memory (Martin et al., 2019). Trait empathy was measured through the Interpersonal Reactivity Index (IRI), a self-report questionnaire in which people were asked to rate the degree to which they agreed with a series of statements. The IRI is developed as a four-dimension scale: perspective taking (PT) measures the ability to shift to another person’s perspective, empathic concern (EC) measures other-oriented feelings of concern for others, fantasy (FS) measures the tendency to become imaginatively absorbed in the feelings and actions of characters in books and movies, and personal distress (PD) measures self-oriented feelings of personal distress caused by the emotions of others. However, Wang et al.’s (2020) study found that better scoring approaches of the IRI include reporting the score of PT as cognitive empathy and the score of EC as emotional empathy. Therefore, we use PT as the cognitive component of empathy, EC as the emotional component of empathy, and total score as trait empathy. Experimentally, state emotional empathy was measured by the Multifaceted Empathy Test (MET, Dziobek et al., 2008; Pang et al., 2022), in which participants respond to an empathy induction designed to elicit a temporary state of emotional empathy.

In summary, cognitive and emotional empathy is taxing. People with higher cognitive flexibility are likelier to regulate emotions effectively and report lower state emotional empathy and trait emotional empathy. Since cognitive flexibility implies more efficient information processing, individuals with higher cognitive flexibility may spend less time on cognitive empathy, report lower trait cognitive empathy, and perform better on state cognitive empathy. The following two research hypotheses were proposed: cognitive flexibility traits and perspective-switching flexibility could negatively predict empathy (Hypothesis 1). Furthermore, since the cognitive flexibility trait reflects the general cognitive tendency, and the perspective switching flexibility reflects the cognitive flexibility in specific cognitive processes, they belong to different levels. Therefore, cognitive flexibility traits and perspective-switching flexibility could still predict empathy even after controlling for one of the two items (Hypothesis 2).

## 2. Measures and methods

### 2.1. Participants

Mainland Chinese participants were drawn from a college in Hebei Province, ages 18 to 23; all participants completed an online questionnaire after completing the laboratory task. The sample was composed of 100 and 20 college students. Fifteen of them were excluded because they got one of the experimental task’s accuracies of less than 80% (the trials in each condition are greater than 15)—the rest of the participants, mean age = 20.26 years, SD = 2.00, 20 of them were male.

### 2.2. Measures

#### 2.2.1. Cognitive flexibility at the individual level

The Chinese version revised by Qi et al. (2013) from the original English version (Martin and Rubin, 1995) was used to measure cognitive flexibility traits. The cognitive flexibility trait scores range from 13 to 78, with higher scores indicating higher cognitive flexibility. The scale consists of 13 items, each of which is a 6-point Likert scale ranging from 1 (strongly disagree) to 6 (strongly agree). Item examples are, “I have many possible ways of behaving in any given situation…” Cronbach’s α for the cognitive flexibility scale in this study was 0.805, similar to those reported in previous studies (range = 0.730–0.870; Martin and Anderson, 1998; Qi et al., 2013).

#### 2.2.2. Cognitive flexibility at the cognitive level

**Figure 1:**
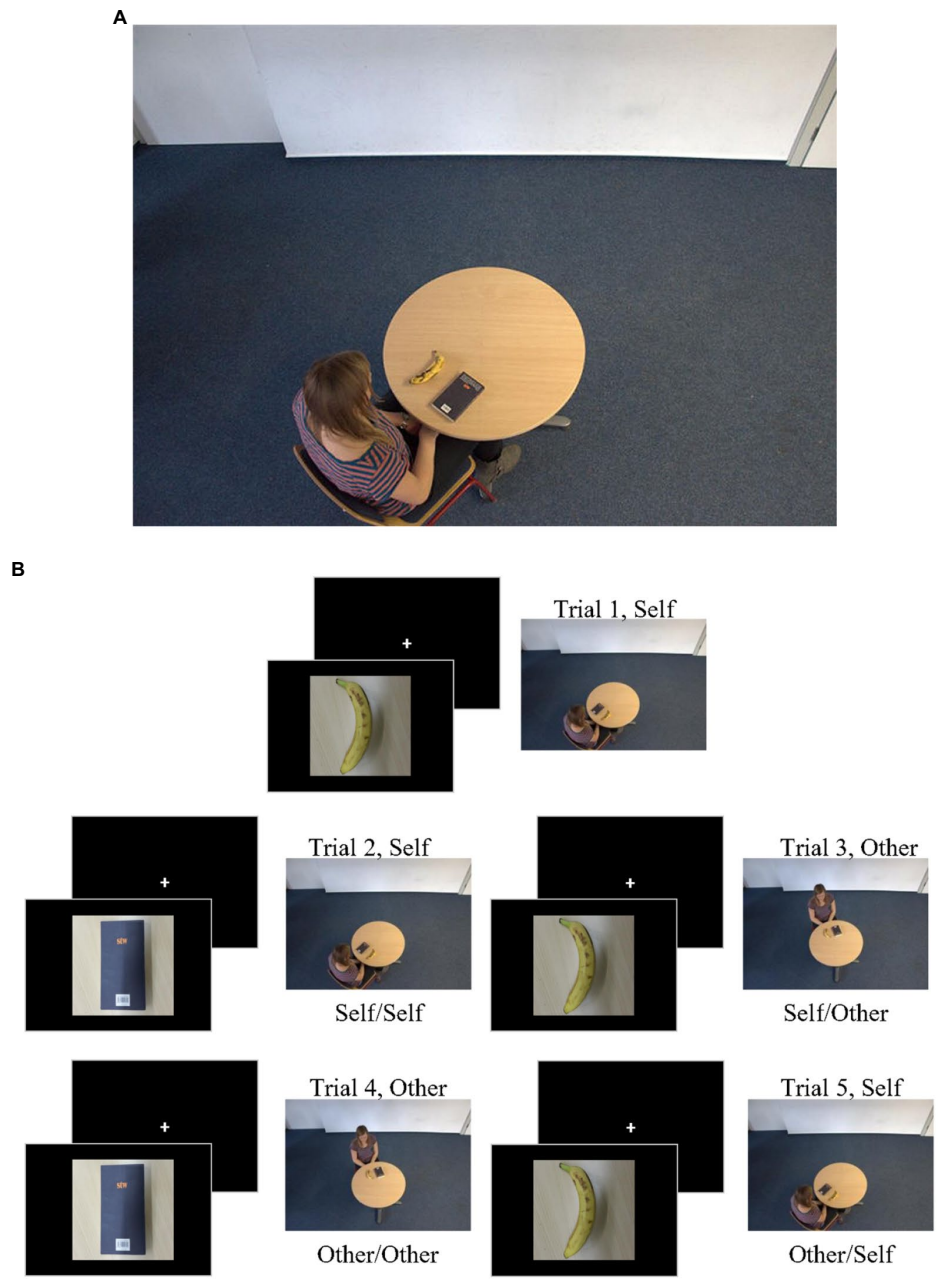
**(A)** A example of level-2 spatial perspective taking. **(B)** The trial sequence of the perspective switching task. The pictures are reproduced with permission from the study of Erle and Topolinski (2017).

An adapted level-2 spatial perspective-taking task was used to measure the perspective-switching flexibility. In the level-2 spatial perspective-taking task, participants were asked to judge the spatial relationship of objects from the perspective of self or persons different from the self. The participants always saw one of two target persons (a man and a woman) sitting at a table with two objects, a book and a banana. One of the objects was target, and participants had to respond with a button which hand (left hand or right hand) the target person would use to pick it from his or her perspective (see Figure 1A).

The angular disparity between the participant and the target is below 80°, the view from self and person has the same position relationship, so the participants judge from self-perspective; The angular disparity between the participant and the target is above 80°, the view from self and person have a different position relationship, so the participants judge from others-perspective (Kessler and Thomson, 2010; Erle and Topolinski, 2017; Xie et al., 2018). In self-perspective condition, the target person sat either at clockwise 40° or 320°. In the others-perspective condition, the target person sat either at clockwise 160° or 240°.

The experimental materials are from public projects see https://osf.io/m92rv. In the perspective-switching task (see Figure 1B), the order of the trials is fixed (two perspectives alternated every two trials, i.e., self-self-other-other-self-self-other-other; Rogers and Monsell, 1995). This sequence of trials allows the perspectives of the current and previous trials was repeating or switching. There are 8 training trials and 81 formal trials. In the repetition condition, there were 40 trials, and participants responded to the current trial were the same perspective as in the previous trial (e.g., self-perspective following self-perspective, other-perspective following other-perspective). In the switching condition, there are 40 trials, and participants responded to the current trial differently from the previous trial (e.g., self-perspective following other-perspective, other-perspective following self-perspective). The reaction time cost between the switch condition and the self-perspective repetition condition reflects the cost of the perspective switch. There are two repetition conditions, Self/Self perspective-repetition for 20 trials and Other/Other perspective-repetition for 20 trials. There are two perspective-switching, Self/Other perspective-switching Other/Other perspective-repetition and Other/self perspective-switching for 20 trials; a smaller cost suggests a faster perspective-switching.

#### 2.2.3. Traits empathy

The IRI measures different components of empathy (Davis, 1980). The 22 items are clustered into four subscales, namely, perspective taking (PT, five items), fantasy (FS, five items), personal distress (PD, six items), and empathic concern (EC, six items). Each item is rated on a 5-point Likert scale from 0 (does not describe me very well) to 4 (describes me very well). Item examples are, “Before criticizing somebody, I try to imagine how I would feel if I were in their place.”(i.e., IRI 22 for PT), “When I am reading an interesting story or novel, I imagine how I would feel if the events in the story were happening to me.”(i.e., IRI 20 for FS), “When I see someone who badly needs help in an emergency, I go to pieces.”(i.e., IRI 21 for PD), “When I see someone being taken advantage of, I feel kind of protective toward them.”(i.e., IRI 7 for EC). The total score of each subscale of IRI ranges from 0 to 24, with a higher score reflecting a stronger trait in the corresponding category. Cronbach’s α of the IRI was 0.773. These values of Cronbach’s α were similar to those reported in previous studies (range = 0.68–0.78; Davis, 1980; Wang et al., 2013).

#### 2.2.4. State empathy

The MET is a computer-based, German-language task that utilizes photographic stimuli for measuring cognitive and emotional empathy within the same task paradigm (MET; Dziobek et al., 2008). There are 40 photographic images of emotional scenes, half positive/pleasant and half negative/unpleasant. All images were randomly presented twice, once to assess cognitive empathy and once to assess emotional empathy. For the cognitive empathy part of the test, participants were asked to select the emotional state of the depicted person from a set of four possible answers by pressing a button. For the emotional empathy part of the test, participants were asked to rate how strongly they experienced the feelings of the person in the picture (i.e., respond to the question “How much do you feel what this person is feeling?”) using a 9-point Likert scale, ranging from 1 (not at all) to 9 (“very strongly”). Where the cognitive empathy score is the sum of the number of correct responses, the emotion empathy score is the sum of the score of the corresponding emotion item.

Based on the English version of the MET, the Chinese translation version of MET Cronbach’s *α* = 0.94, show good validity and reliability and can be used to measure the empathy of Chinese participants (Pang et al., 2022). With this study sample, the Cronbach’s *α* for the total scale was 0.83.

#### 2.2.5. Inhibition control

In order to measure the participant’s inhibitory control, a Stroop task was employed. The task contained 8 training trials and 40 formal trials. In each trial, a fixation would be randomly present in the Centre screen during 500–800 ms, then a red or green “red” or “green.” Then, the participants were required to make a reaction within 3,000 ms. In this task, the participants had to judge the color of the words appearing on the screen response by button (“D “or “K “). There were 20 consistent and inconsistent trials. The red “red” and the green “green” were consistent trials, and the green “red” and the red “green” were inconsistent trials. The quotient of the inconsistent over consistent condition was the index of the inhibitory control score, where larger quotient indicated poorer inhibitory control.

#### 2.2.6. Working memory

Working memory was measured with 2-back task. There are 8 training trials and 44 formal trials. The stimulus material consisted of 10 digits (0–9). In each trial, a digit would be presented 500 ms, and the participants were required to make a reaction within 3,000 ms. The participants were required to compare the current digit to the second one preceding the digit, pressing “C” if it matched and pressing “M” if it disagreed. The order of pressing the button was balanced between participants. For example, the digit sequence is “1, 3, 1, 2,” and participants were required to make consistent judgments about the third digit (“1”) and inconsistent judgments about the fourth digit (“2”). There were 22 trials each for the congruent and incongruent conditions. It used E Prime software, stimulus presentation and recording of task responses. The accuracy was calculated using arcsine square root of the proportion of correct responses to make the data conform to a normal distribution (Martin et al., 2019), and the converted accuracy was used as the working memory score.

### 2.3. Procedure

Study invitations were randomly distributed on campus. All participants were asked to read the study introduction and fill out personal demographic information before participating. First, four tasks (perspective-switching task, 2-back task, MET, and Stroop task) were completed on the computer in the laboratory. Each task took five to 8 minutes, with a three-minute break. Then two questionnaires (IRI-C and CFS) were filled in online. If no questions were answered, the survey could not be submitted, so no data was missed.

### 2.4. Data analysis

Excel was used to complete the data preprocessing, the extreme data less than 100 ms and more than 3,000 ms were excluded, and then eliminating the trials outside M ± 2.5SD for each condition in the laboratory task (perspective-switching task and Stroop task). These trials account for 3.10% of the total data. Then, we examined whether the manipulation of experimental conditions was valid using independent-sample *t*-tests. Harman’s single-factor test was used for common method bias in self-reported data. Preliminary descriptive analyses included *t*-tests for condition differences in the study task, Cohen’s *d* as a measure of *t*-test effect size, and Pearson’s bivariate correlation.

According to the study’s aims, we ran two regression models to predict empathy. In the first kind of model, the focal predictor was cognitive flexibility traits. Gender, age, inhibition control, working memory, and cognitive flexibility at the cognitive level were covariate variables. In contrast, in the second kind of model, the focal predictor was perspective-switching flexibility. Gender, age, inhibition control, working memory, and cognitive flexibility at the individual level were the covariate variables.

### 2.5. Results

The results of paired sample *t*-test showed that in the perspective-switching flexibility task, the Self/Other perspective-switching cost/accuracy (*M*_RT_ = 275, SD = 188; *M*_ACC_ = 92.33%, SD = 10.17%) was significantly longer/lower than Other/Self perspective-switching (*M*_RT_ = 62, SD = 60, *t*(104) = 12.236, *p* ≤ 0.001, Cohen’s *d* = 1.526; *M*_ACC_ = 96.29%, SD = 5.55%, *p* ≤ 0.001, Cohen’s *d* = 0.483). In the Stroop task, the response time/accuracy of the inconsistent condition (*M*_RT_ = 621, SD = 133; *M*_ACC_ = 93.10%, SD = 18.09%) was significantly longer/lower than that of the consistent condition (*M*_RT_ = 569, SD = 110, *t*(104) = 8.632, *p* ≤ 0.001, Cohen’s *d* = 0.426; *M*_ACC_ = 94.57%, SD = 18.91%, *p* ≤ 0.001, Cohen’s *d* = 0.008). Since the response was slower when the accuracy was lower and faster when the accuracy was higher, suggesting that there was no trade-off between the accuracy and the reaction time. It indicates that the manipulation of different conditions of each task is effective.

#### 2.5.1. Common method variance

This study included self-reported data, which may be subject to common method bias. In the process of data collection, the questionnaire included reverse scoring questions. In the statistical analysis of the data, the Harman’s single factor test was used to test the common method deviation. The results of Harman test showed that there were 12 factors with eigenvalues greater than 1, which explained 70.33% of the variation. The first factor explained 17.517% of the variation, which was less than 40% of the critical value. This indicates that there is no common method bias in the questionnaire measurement data (Xiong et al., 2012).

#### 2.5.2. Descriptive and regression analysis

Means, Standard Deviations, and the correlation coefficients (the top right corner of the table controls for gender, age, inhibitory control, and working memory) are reported in Table 1. The correlation coefficients reveal that students with high cognitive flexibility traits had lower scores in PT (*r* = −0.450, *p* < 0.001), EC (*r* = −0.359, *p* < 0.001), trait empathy (*r* = −0.244, *p* = 0.014) and higher scores in PD (*r* = 0.211, *p* = 0.034). Other/Self perspective-switching cost had a negative correlation with the emotional component of state empathy (*r* = 0.279, *p* = 0.005). Since the correlation coefficients between cognitive flexibility trait and state empathy and perspective-switching flexibility and trait empathy were not significant, no further linear regression results were reported.

**Table 1:** Correlation coefficients of empathy, and cognitive flexibility.

	*M*	SD	1	2	3	4	5	6	7	8	9	10
1 PT	12.80	3.39	1	0.348^***^	0.061	0.457^***^	0.372^***^	−0.135	0.047	−0.450^***^	0.041	0.005
2 FS	16.07	2.82	0.316^**^	1	0.296^**^	0.216^*^	0.701^***^	−0.188	0.057	−0.050	0.137	0.062
3 PD	14.56	3.27	0.14	0.340^**^	1	0.055	0.559^***^	−0.030	0.049	0.211^*^	0.131	0.070
4 EC	17.85	2.61	0.386^**^	0.180	0.036	1	0.623^***^	−0.147	0.017	−0.359^***^	−0.036	−0.026
5 TE	61.28	7.73	0.690^**^	0.709^**^	0.566^**^	0.588^**^	1	−0.186	0.066	−0.244^*^	0.073	0.044
6 CSE	23.10	2.98	−0.099	−0.187	−0.052	−0.160	−0.188	1	0.080	0.004	0.092	0.116
7 ESE	220.34	41.04	0.072	0.028	0.018	−0.046	0.034	0.080	1	−0.045	0.148	0.279^**^
8 CFT	37.67	7.03	−0.468^**^	−0.017	0.257^**^	−0.339^**^	−0.217^*^	−0.020	−0.049	1	0.099	0.112
9 S/O–PS	62	60	−0.054	0.171	0.165	−0.023	0.101	0.059	0.124	0.110	1	0.295^**^
10 O/S–PS	275	188	0.018	0.094	0.088	−0.041	0.066	0.117	0.254^**^	0.104	0.312^**^	1

PT, Perspective Taking; FS, Fantasy; PD, Personal Distress; EC, Empathic Concern; CSE, the Cognitive component of State Empathy; ESE, the Emotional component of State Empathy; CFT, Cognitive Flexibility Trait; S/O-PS, Self/Other Perspective-Switching; O/S-PS, Other/Self Perspective-Switching. *p < 0.050; **p < 0.010; ***p < 0.001.

We performed regression analysis to verify whether the associations of cognitive flexibility trait and trait empathy could be observed when demographics, inhibition control, working memory, and flexibility in perspective-switching were controlled for. In model 1, predictor variables were demographics, inhibition control, and working memory. In model 2, the predictive variables were based on Model 1 with the addition of perspective-switching flexibility. The predictive variables of model 3 included Model 2 and cognitive flexibility traits. According to the PT that can be found in Table 2, the increasing variance of models 1 (Δ*R*^2^ = 0.051, *p* = 0.258) and 2 (Δ*R*^2^ = 0.002, *p* = 0.908) was not significant. Model 3 significantly increased the variance by 19.4% (Δ*R*^2^ = 0.194, *p* ≤ 0.001), and cognitive flexibility traits (*β* = −0.456, *t* = −4.991, *p* ≤ 0.001) showed negative beta coefficients for PT. For the EC shown in Table 3, the increasing variance of models 1 (Δ*R*^2^ = 0.099, *p* = 0.032) and 3 (*ΔR*^2^ = 0.115, *p* ≤ 0.001) is significant. In model 3, gender (*β* = −0.303, *t* = −3.289, *p* ≤ 0.001) and cognitive flexibility traits (*β* = −0.352, *t* = −3.773, *p* ≤ 0.001) showed statistically significant and negative beta coefficients for EC. Table 4 shows the dependent variable was PD, the increasing variance of model 1 (Δ*R*^2^ = 0.060, *p* = 0.037) and 3 (Δ*R*^2^ = 0.086, *p* = 0.049) is significant. In model 3, cognitive flexibility traits (*β* = 0.194, *t* = 1.994, *p* = 0.049) showed statistically significant and positive beta coefficients for PD. When trait empathy is the dependent variable, the results are summarized in Table 5. As we can see, the *R*^2^ of model 3 (*R*^2^ *=* 0.097, *F* = 1.497, *p* = 0.177) is larger than that of model 1 (*R*^2^ *=* 0.028, *F* = 0.726, *p* = 0.576) and 2 (*R*^2^ *=* 0.034, *F* = 0.574, *p* = 0.756), and statistically significantly increased the variance by 6.4% (*ΔR*^2^ = 0.064, *p* = 0.010). In model 3, the cognitive flexibility trait (*β* = −0.261, *t* = −2.613, *p* = 0.010) significantly negatively predicted trait empathy.

**Table 2:** Result of linear regression analysis with PT as the dependent variable.

	*β*	SE	*t*	*F*	*R* ^2^	Δ*R*^2^
Step 1						
Gender	0.195	0.976	1.987^*^	1.348	0.051	0.051
Age	0.050	0.178	0.508			
Inhibition control	0.117	3.089	1.195			
Working memory	−0.061	1.057	−0.621			
Step 2						
Gender	0.189	0.995	1.889	0.915	0.053	0.002
Age	0.043	0.183	0.425			
Inhibition control	0.121	3.140	1.216			
Working memory	−0.061	1.007	−0.608			
S/O-PS	−0.061	0.002	−0.436			
O/S-PS	−0.047	0.006	0.173			
Step 3						
Gender	0.148	0.896	1.646	4.534^***^	0.247	0.194^***^
Age	−0.015	0.165	−0.160			
Inhibition control	0.040	2.861	0.447			
Working memory	−0.035	0.967	−0.384			
S/O-PS	−0.013	0.002	−0.138			
O/S-PS	0.059	0.005	0.625			
CFT	−0.456	0.044	−4.991^**^			

*p < 0.050; **p < 0.010; ***p < 0.001.

**Table 3:** Result of linear regression analysis with EC as the dependent variable.

	*β*	SE	*t*	*F*	*R* ^2^	Δ*R*^2^
Step 1						
Gender	−0.268	0.731	−2.800^***^	2.755^*^	0.099	0.099
Age	0.161	0.133	1.692			
Inhibition control	0.045	2.314	0.468			
Working memory	0.001	0.792	−0.003			
Step 2						
Gender	−0.271	0.746	−2.778^***^	1.828	0.101	0.001
Age	0.154	0.137	1.574			
Inhibition control	0.049	2.353	0.507			
Working memory	−0.004	0.807	−0.042			
S/O-PS	−0.030	0.001	−0.292			
O/S-PS	−0.016	0.004	−0.162			
Step 3						
Gender	−0.303	0.703	−3.289^***^	3.812^***^	0.216	0.115^***^
Age	0.110	0.129	1.187			
Inhibition control	−0.013	2.24	−0.139			
Working memory	0.016	0.759	0.177			
S/O-PS	−0.005	0.001	−0.048			
O/S-PS	0.015	0.004	0.155			
CFT	−0.352	0.035	−3.773^**^			

*p < 0.050; **p < 0.010; ***p < 0.001.

**Table 4:** Result of linear regression analysis with PD as the dependent variable.

	*β*	SE	*t*	*F*	*R* ^2^	Δ*R*^2^
Step 1						
Gender	−0.109	0.918	−1.139	2.667^*^	0.096	0.096^*^
Age	−0.227	0.167	−2.378^*^			
Inhibition control	−0.163	2.906	−1.708			
Working memory	−0.082	0.994	−0.858			
Step 2						
Gender	−0.096	0.928	−0.994	2.077	0.113	0.016
Age	−0.202	0.170	−2.077^*^			
Inhibition control	−0.179	2.930	−1.856			
Working memory	−0.072	1.005	−0.739			
S/O-PS	0.119	0.002	1.150			
O/S-PS	0.034	0.006	0.332			
Step 3						
Gender	−0.079	0.918	−0.824	2.403^*^	0.148	0.035^*^
Age	−0.178	0.169	−1.839			
Inhibition control	−0.144	2.933	−1.499			
Working memory	−0.083	0.992	−0.866			
S/O-PS	0.105	0.002	1.026			
O/S-PS	0.016	0.005	0.163			
CFT	−0.194	0.045	1.994^*^			

*p < 0.050.

**Table 5:** Result of linear regression analysis with trait empathy as the dependent variable.

	*β*	SE	*t*	*F*	*R* ^2^	Δ*R*^2^
Step 1						
Gender	−0.063	2.250	−0.632	0.726	0.028	0.028
Age	−0.082	0.410	−0.832			
Inhibition control	−0.021	7.120	−0.210			
Working memory	−0.118	2.436	−1.186			
Step 2						
Gender	−0.056	2.289	−0.551	0.547	0.034	0.006
Age	−0.068	0.420	−0.667			
Inhibition control	−0.030	7.223	−0.299			
Working memory	−0.111	2.477	−1.098			
S/O-PS	0.067	0.004	0.624			
O/S-PS	0.025	0.014	0.237			
Step 3						
Gender	−0.079	2.233	−0.802	1.497	0.097	0.064^**^
Age	−0.101	0.411	−1.011			
Inhibition control	−0.076	7.132	−0.768			
Working memory	−0.096	2.411	−0.975			
S/O-PS	0.086	0.004	0.823			
O/S-PS	0.048	0.013	0.469			
CFT	−0.261	0.110	−2.613^**^			

**p < 0.010.

Table 6 summarizes the regression model that when demographics, inhibition control, working memory, and cognitive flexibility trait were controlled for, the relationship between perspective-switching cost and the emotional component of state empathy. In model 1, predictor variables were demographics, inhibition control, and working memory. In model 2, the predictive variables were based on Model 1 with the addition of cognitive flexibility traits. The predictive variables of model 3 included Model 2 and perspective switching flexibility. Model 3 (*R*^2^ = 0.160*, F* = 2.647*, p* = 0.015), which includes the flexibility of perspective switching, significantly increased the variance by 8% (Δ*R*^2^ = 0.080, *p* = 0.012) and is better than models 1 (*R*^2^ = 0.078, *F* = 2.116, *p* = 0.084) and 2 (*R*^2^ = 0.080*, F* = 1.720*, p* = 0.137). In model 3, the working memory positively predicts the emotional component of state empathy (*β* = 0.220, *t* = 2.319, *p* = 0.022), and the flexibility of Other/Self perspective-switching costs negative prediction the emotional component of state empathy (*β* = 0.259, *t* = 2.608, *p* = 0.011).

**Table 6:** Result of linear regression analysis with the emotional component of state empathy as the dependent variable.

	*β*	SE	*t*	*F*	*R* ^2^	Δ*R*^2^
Step 1						
Gender	0.180	0.291	1.853	2.116	0.078	0.078
Age	−0.092	0.053	−0.955			
Inhibition control	0.054	0.921	0.557			
Working memory	0.177	0.315	1.828			
Step 2						
Gender	0.175	0.294	1.793	1.720	0.080	0.02
Age	−0.099	0.054	−1.839			
Inhibition control	0.046	0.937	−0.451			
Working memory	0.179	0.317	3.099			
Cognitive flexibility trait	−0.045	0.014	1.809			
Step 3						
Gender	0.172	0.286	1.809	2.647^*^	0.160	0.080^*^
Age	−0.067	0.053	−0.695			
Inhibition control	0.014	0.913	0.149			
Working memory	0.220	0.309	2.319^*^			
Cognitive flexibility trait	−0.082	0.014	−0.846			
S/O-PS	0.077	0.001	0.765			
O/S-PS	0.259	0.002	2.608^*^			

*p < 0.050.

## 3. Discussion

### 3.1. The relationship between cognitive flexibility and empathy

This study extends the research on cognitive flexibility and empathy by exploring the relationship between different levels of cognitive flexibility and empathy.

In general, the results of correlation analysis showed that cognitive flexibility traits negatively predicted traits of empathy (PT as the trait of cognitive empathy, EC as the trait of affective empathy, and total score of IRI as traits of empathy), and perspective-switching flexibility negatively predicted the emotional component of state empathy, supporting hypothesis 1. Empathy is felt as cognitively costly, even a less obvious cost that can motivate people to avoid empathy. According to Cameron et al. (2019), positive emotional empathy for others also has costs. Hence, the cognitive system effectively processing information and regulating emotion is essential. Cognitive flexibility is a property of the cognitive system, and people with higher cognitive flexibility have a better cognitive system. As a multi-level structure, cognitive flexibility manifests at both the cognitive and individual levels. People with higher cognitive flexibility also have better interactions between the cognitive systems (e.g., executive functions, attentional mechanisms, representations) and between the cognitive system and the environment (e.g., interaction of cognition, task demands, and contextual cues). At the cognitive level, cognitive flexibility means faster information processing and better attention switching (Monsell et al., 2003). It also shows the ability to flexibly reorganize cognition according to their goals and environment (Braem and Egner, 2018). At the individual level, the cognitive flexibility trait reflects the consciousness, willingness, and efficacy of individuals to make flexible choices in the face of the environment (Martin and Rubin, 1995), which is not only related to emotion regulation ability (Gao et al., 2021) and adaptation to the environment (Wang and Liu, 2017). This suggests that cognitive flexibility can play a more influential role in the cognitive system and emotional regulation that reduced the empathic response and scored lower on the trait’s cognitive empathy and emotional empathy (trait and state emotional empathy). The results may be explained by the fact that cognitively flexible people have a worse cognitive ability for empathy. However, the cognitive flexibility trait was not associated with behavior-based empathy, suggesting that people with cognitive flexibility may process others’ information more efficiently and pay less attention to others. At the cognitive level, better flexibility in perspective allows people to process themselves and other people’s perspectives better. The results are consistent with the cognitive flexibility trait, showing that individuals who are more flexible in Other/Self perspective-switching are less likely to feel the emotions of others. This suggests that the more flexible the perspective-shifting, the more able they can regulate the process of automatic emotional responses to others.

Interestingly, Thoma et al. (2011) found that cognitive flexibility (measured by Trail Making Test) was positively associated with state cognitive empathy. Different results from this study may be because the participants in their study were depressive disorders, had cognitive and emotional processing deficits, and had low levels of cognitive flexibility that could only support them to empathize without being able to adjust cognition flexibly to the environment effectively. Our study found a negative correlation between cognitive flexibility traits and the cognitive component of trait empathy (IRI-PT). Participants with higher cognitive flexibility traits suggest they have the consciousness, willingness, and efficacy to make flexible choices in any situation. Hence, as cognitive flexibility develops, there may be a reversal in the relationship between cognitive flexibility and empathy. It may also be because the tools used to measure cognitive flexibility are different.

Meanwhile, even after controlling for one item, the negative predictive effect of cognitive flexibility trait or perspective-switching flexibility on empathy was still significant, supporting hypothesis 2. This indicates that individual and cognitive flexibility are two different levels of flexibility (Ionescu, 2012). The former reflects the general tendency of individuals to interact with the environment, while the latter reflects the flexibility of individuals in specific cognitive processes. In addition, working memory was found to positively predict empathy, consistent with previous findings (Yan et al., 2020), indicating that executive function as a component of cognitive flexibility plays a vital role in empathy.

Finally, we did not find that the cognitive flexibility trait or perspective-switching flexibility negatively predicted PD. Unlike other-oriented EC, PD is a self-oriented negative emotional response that may bring potential emotional stress to people and affect their mental health (Yan et al., 2022). Since PD reflects the ability to perceive others’ painful emotions, it is similar to emotional contagion and is a relatively automatic process. Cognitive flexibility might allow one to feel others’ distress but be able to switch them off if needed.

### 3.2. Limitations and future research

Although the study controlled for some additional variables, other factors not tested here may be relevant to empathic processes, such as interoceptive ability and alexithymia. Interoception refers to the detection and perception of signals from the inner body, contributing to inferences about the affective state of others (Fukushima et al., 2011). Alexithymia (a difficulty identifying and expressing emotions experienced by oneself or others) occurs in approximately 10% of the general population (Mattila et al., 2006) and is related to decreased empathic behaviors (Grynberg et al., 2010). Future research can explore the possible moderating or mediating role of Alexithymia and Interoception to clarify the mechanism by which cognitive flexibility plays a role in empathy. In addition, this study could not conclude a direct causal relationship between cognitive flexibility and empathy. Future research can use techniques such as experiments or interventions to explore the impact of cognitive flexibility on empathy.

## Data availability statement

The raw data supporting the conclusions of this article will be made available by the authors, without undue reservation.

## Ethics statement

The studies involving human participants were reviewed and approved by College of Education, Hebei University. The patients/participants provided their written informed consent to participate in this study.

## Author contributions

ZC and BQ contributed to conception and design of the study and wrote sections of the manuscript. ZC organized the database, performed the statistical analysis, and wrote the first draft of the manuscript. All authors contributed to the article and approved the submitted version.

## Conflict of interest

The authors declare that the research was conducted in the absence of any commercial or financial relationships that could be construed as a potential conflict of interest.

## Publisher’s note

All claims expressed in this article are solely those of the authors and do not necessarily represent those of their affiliated organizations, or those of the publisher, the editors and the reviewers. Any product that may be evaluated in this article, or claim that may be made by its manufacturer, is not guaranteed or endorsed by the publisher.
